# Global cardiovascular risk profiles of untreated hypertensives in an urban, developing community in Africa

**DOI:** 10.5830/CVJA-2010-094

**Published:** 2011-10

**Authors:** Muzi J Maseko, Gavin R Norton, Olebogeng HI Majane, Nomonde Molebatsi, Angela J Woodiwiss

**Affiliations:** Cardiovascular Pathophysiology and Genomics Research Unit, School of Physiology, Faculty of Health Sciences, University of the Witwatersrand, Johannesburg, South Africa; Cardiovascular Pathophysiology and Genomics Research Unit, School of Physiology, Faculty of Health Sciences, University of the Witwatersrand, Johannesburg, South Africa; Cardiovascular Pathophysiology and Genomics Research Unit, School of Physiology, Faculty of Health Sciences, University of the Witwatersrand, Johannesburg, South Africa; Cardiovascular Pathophysiology and Genomics Research Unit, School of Physiology, Faculty of Health Sciences, University of the Witwatersrand, Johannesburg, South Africa; Cardiovascular Pathophysiology and Genomics Research Unit, School of Physiology, Faculty of Health Sciences, University of the Witwatersrand, Johannesburg, South Africa

**Keywords:** blood pressure control, antihypertensive treatment, detection of hypertension

## Abstract

**Introduction:**

Blood pressure (BP) control in people of African descent is poor, largely because of a lack of treatment. Although the requirements for immediate initiation of antihypertensive drug therapy are defined by global cardiovascular risk, the global cardiovascular risk profiles of untreated hypertensives at a community level are uncertain.

**Aim:**

To identify the distribution of global cardiovascular risk profiles of untreated hypertensives in an urban, developing community of African descent in South Africa.

**Methods:**

As part of the African Programme on Genes in Hypertension, we assessed nurse-derived clinic BP (the mean of five standardised BP values obtained according to guidelines), current antihypertensive therapy, and total cardiovascular risk in 1 029 participants older than 16 years of age from randomly selected nuclear families from the South West Township of Gauteng (SOWETO).

**Results:**

Approximately 46% of participants had systolic/diastolic BP values ≥ 140/90 mmHg and ~23% of participants were hypertensives not receiving antihypertensive medication. Approximately 12% of untreated hypertensives had a high added risk and ~18% a very high added risk (6.7% of the total sample). In untreated hypertensives, in contrast to the absence of severe hypertension and diabetes mellitus in those with lower risk profiles, a high cardiovascular risk profile in this group was characterised by severe hypertension in ~52% and diabetes mellitus in ~33%. Based on a high added risk carrying at least a 20% chance and a very high added risk at least a 30% chance of a cardiovascular event in 10 years, this translates into 1 740 events per 100 000 of the population within 10 years, events that could be prevented through antihypertensive drug therapy.

**Conclusions:**

In an urban, developing community of African ancestry, a significant proportion (6.7%) of people may have untreated hypertension and a global cardiovascular risk profile that suggests a need for antihypertensive drug therapy. Cardiovascular risk in this group is driven largely by the presence of severe hypertension or diabetes mellitus.

## Summary

In economically developed countries, there is a continuous relationship between blood pressure (BP) and the risk of mortality from ischaemic heart disease and stroke,^1^ and hypertension is the second most common risk factor for end-stage renal disease.^2^ In these countries, the risk for cardiovascular disease exists in both elderly and young (18–39 years old) hypertensives.^3^

In economically emerging countries, hypertension has materialised as the most prevalent risk factor for heart failure,^4^ coronary artery disease,^5^ and stroke.^6^,^7^ In contrast to the approximately 34–35% of all hypertensives and 55% of treated hypertensives that are controlled to target BP levels in economically developed countries,^8^–^11^ by comparison, in economically emerging countries such as South Africa, only 14% of all hypertensives^12^ and 33–44% of treated hypertensives in primary-care settings^13^,^14^ are controlled to target BP. The major barrier to BP control in economically emerging countries is lack of treatment.^12^

Although a lack of treatment accounts for inappropriate BP control in economically emerging communities,^12^ the decision to treat hypertension with drug therapy should be based on global cardiovascular risk scores, rather than on the presence of hypertension *per se*.^15^ In this regard, it is acknowledged^16^ that there is little evidence to support the use of drug therapy in people who have BP values between 140 and 159/90 and 99 mmHg and a ‘low or moderate added’ risk. Indeed, the most recent evidence suggesting the use of drug therapy in this category is derived from a study where 89% of patients were already receiving antihypertensive therapy at baseline.^17^

As BP control in the context of global cardiovascular risk in untreated hypertensives in emerging communities in Africa has to our knowledge never been described, in the present study we aimed to determine the cardiovascular risk profiles of untreated hypertensives in an urban, developing community of African descent.

## Methods

The present ongoing cohort study, which has been described in recent publications,^18^–^25^ was initiated in 2002 and conducted according to the principles outlined in the Helsinki declaration. The Committee for Research on Human Subjects of the University of the Witwatersrand approved the protocol (approval number: M02-04-72 and renewed as M07-04-69). Participants gave informed, written consent. Nuclear families of black African descent (Nguni and Sotho chiefdoms) with siblings older than 16 years were randomly recruited from the South West Township (SOWETO) of Johannesburg, South Africa, using the population census figures of 2001. From the time of initiation of the study until the end of 2009, 1 029 participants have been studied.

A standardised questionnaire^18^-^25^ was administered to obtain demographic and clinical data. In order to avoid translational errors, the questionnaire was not translated into an African language, but study assistants familiar with all languages spoken in SOWETO and who either previously lived in or currently reside in SOWETO assisted with the completion of each questionnaire. However the majority of participants were reasonably proficient in English.

Only same-sex assistants were used to assist each family member with the completion of the questionnaire. Assistance was provided only when requested. Study assistants first visited homes of subjects who agreed to participate in the study in order to familiarise participants with the questionnaire. The questionnaire was completed at a subsequent clinic visit and then ambiguities were checked by performing a follow-up home visit. If family members were absent at follow-up home visits, data were checked with them personally via telephonic conversations whenever possible. Ambiguities in answers to the questionnaire were detected by an independent observer prior to a second home visit. A pilot study was conducted in 20 participants to ensure that data obtained in the questionnaires were reproducible when obtained with the assistance of two separate study assistants.

The questionnaire requested specific answers to date of birth, gender, previous medical history (including the presence of hypertension, diabetes mellitus and kidney disease), previous cardiovascular events (including stroke, myocardial infarction or heart failure), the presence of angina pectoris, prior and current drug therapy (analgesic, antihypertensive use and glucose lowering agents included), smoking status (including the number of cigarettes smoked in the past and at the present time), daily alcohol consumption (beer, traditional beer or other forms of alcohol and the daily quantity), caffeine consumption (number of cups of tea or coffee and whether they are decaffeinated, and the number of colas a day), exercise frequency, and family history of hypertension. For females, menstrual history, history of pregnancies and use of oral contraceptives was evaluated. Most of the questions simply required a ‘yes’/‘no’ answer, but understanding was assessed by requesting some short answers. If participants were unable to provide the name of medications taken, these were obtained on a second home visit.

High-quality conventional BP measurements were obtained by a study nurse using a standard mercury sphygmomanometer, as previously described.^19^ After being trained in the procedure, including being shown the pitfalls of BP measurement (positioning of the cuff, positioning of the arm, first estimating systolic BP using a radial pulse measure in order to avoid inflating cuff pressures too high, detecting auscultatory gaps, releasing valve pressure at the correct speed, using the correct cuff size, etc) the observer demonstrated an ability to perform the procedure on 20 participants.

The study assistant was able to measure BP on a separate group of 20 participants, including patients with hypertension, to within 4 mmHg of an experienced investigator obtained with a stethoscope with two ear pieces. The study assistant was also able to detect phase I and V sounds under different circumstances, including in the presence of a wide auscultatory gap and where phase V Korotkoff was taken as a ‘muffling’ rather than a ‘disappearance’ of sounds (Blood Pressure Measurement, *British Medical Journal*, BMA House London).

A standard cuff with a 12 × 24-cm inflatable bladder was used to assess conventional BP, but if upper arm circumference exceeded 31 cm, larger cuffs with a 15 × 35-cm inflatable bladder were used. After 10 minutes of rest in the seated position, five consecutive BP readings were taken 30 to 60 seconds apart with the participants in a seated position, followed by a pulse rate count. The cuff was deflated at approximately 2 mmHg per second, and phase I (systolic) and phase V (diastolic) BP was recorded to the nearest 2 mm Hg. The average of the five readings was taken as the office BP.

In the present study, quality control of conventional BP assessments was assessed as previously described.^26^ Only 0.68% of visits had fewer than the planned BP recordings. The frequency of identical consecutive recordings was 0.87% for systolic and 2.24% for diastolic BP. The occurrence of BP values recorded as an odd number was 0%. Of the systolic and diastolic BP readings, 30.1% ended on a zero (expected = 20%).

Body height, weight, waist and hip circumference, and triceps and subscapular skin-fold thickness (Harpenden Skinfold Calliper, Bedfordshire, UK) were measured during the clinic visit by a trained observer. Height and weight were measured with the participants standing and wearing indoor clothes with no shoes. Waist and hip circumferences were measured according to conventional techniques. Body mass index (BMI) was calculated as weight in kilograms divided by the square of height in metres, and waist-to-hip ratio was calculated as an index of central obesity. Participants were identified as being overweight if their BMI was ≥ 25 kg/m^2^ and obese if it was ≥ 30 kg/m^2^. Participants were identified as having central obesity if waist circumference exceeded 102 cm in men and 88 cm in women.

Blood samples were obtained on the day of the visit and the South African National Health Systems Laboratories (NHSL) contract laboratory services (established for scientific studies) performed a full blood count and differential count, measured urea, creatinine and electrolyte concentrations, assessed liver function (from alanine transaminase, aspartate transaminase, gamma glutamyl transpeptidase, alkaline phosphatase, albumin, total protein and plasma albumin, total bilirubin, and conjugated and unconjugated bilirubin concentrations), measured plasma urate concentrations, determined a lipid profile (total cholesterol, and low-density lipoprotein cholesterol, high-density lipoprotein cholesterol and triglyceride concentrations), and performed a blood glucose measurement and percentage glycated haemoglobin (HbA_1c_). A ‘spot’ urine analysis was also performed to screen for major clinical conditions, such as diabetes mellitus (DM) and renal pathology.

Cardiovascular risk was defined using two approaches. Risk was assessed according to the Southern African Hypertension Society (SAHS) guidelines^27^ which are similar to the recently published European Society of Hypertension/European Society of Cardiology (ESH/ESC) guidelines.^16^ These guidelines suggest that antihypertensive treatment should be instituted in people with a high or very high added risk, which equates to an absolute 10-year risk of a cardiovascular event of 20–30% (high added risk) or > 30% (very high added risk).^16^,^27^

Second, cardiovascular risk was defined according to the World Health Organisation and International Society of Hypertension (WHO/ISH) risk prediction charts for low- and middle-income countries.^28^ These guidelines suggest that antihypertensive therapy is instituted when there is an absolute 10-year risk of a cardiovascular event of > 30%.^28^ The WHO/ISH risk charts guidelines exclude an analysis of the impact of adiposity (as well as HDL cholesterol, and a family history of premature CVD), and risk in the 20–40 year age category.

As we previously demonstrated that obesity is highly prevalent and contributes to cardiovascular damage in urban, developing communities in South Africa,^18^–^24^ and we have noted that a significant burden of severe hypertension (BP ≥ 180/110 mmHg) exists in participants younger than 40 years, we focused our analysis on the SAHS/ESH/ESC guidelines which do not discount these factors. With respect to target-organ changes, only plasma creatinine concentrations were employed to identify organ damage, although national guidelines suggest that urinary microalbumin and electrocardiography criteria also be included,^27^ and international guidelines suggest that echocardiography and the assessment of vascular structure and function be included.^16^

The reasons for adopting this approach in the present study are as follows. National guidelines only advocate the measurement of urinary microalbumin in patients with DM.^27^ Moreover, in the present study some participants did not provide urine samples. The high prevalence of obesity (~40%) considerably limited the value of electrocardiographic criteria for left ventricular hypertrophy in this study; and the high prevalence of obesity resulted in echocardiographic and vascular data (aortic pulse-wave velocity) being unavailable in a subset of participants.

The combined evidence for decreasing BP to targets lower than 140/90 mmHg in patients with DM or renal dysfunction is controversial.^29^ Blood pressure was therefore only considered as uncontrolled if the average clinic reading was ≥ 140/90 mmHg even in participants with DM or renal dysfunction. Moderate and severe hypertension or moderate and severe increases in BP values were identified as systolic BP = 160–179 mmHg or diastolic BP = 100–109 mmHg (moderate hypertension) and systolic BP ≥ 180 mmHg or diastolic BP ≥ 110 mmHg (severe hypertension).^16^,^27^ Diabetes mellitus was defined as the use of insulin or oral hypoglycaemic agents, or a fasting blood glucose ≥ 7.0 mmol/l, or a post-prandial glucose ≥ 11.0 mmol/l and an HbA_1c_ value > 6.1%.^30^

## Statistical analysis

For database management and statistical analysis, SAS software, version 9.1 (SAS Institute Inc, Cary, NC) was employed. Data are shown as mean ± SD or percentages. Age adjustments were determined from multivariate regression models and differences in proportions were identified using Fisher’s exact test.

## Results

Table 1 gives the characteristics of the normotensive participants, the hypertensive patients not receiving therapy, and the hypertensive patients receiving therapy; 23.5% of the participants were hypertensives receiving antihypertensive drug therapy and 22.6% were hypertensives not receiving therapy. Both untreated and treated hypertensives were older, and had an increased BMI, waist circumference and waist-to-hip ratio. A greater proportion of untreated and treated hypertensives were obese, and either receiving therapy for DM or had a fasting blood glucose ≥ 7.0 mmol/l or a post-prandial blood glucose ≥ 11.0 mmol/l and an impaired blood glucose control (HbA_1c_ > 6.1%).

**Table 1.** Characteristics Of Study Participants

	*Normotensives*	*Untreated hypertensives*	*Treated hypertensives*
Number	554	233	242
Age (years)	33.8 ± 14.5	50.4 ± 15.9*	60.9 ± 11.9*
% female	66.3	55.4	74.0
Body mass index (kg/m^2^)	27.1 ± 7.4	30.6 ± 7.5*	33.8 ± 7.9*
% overweight/obese	21.7/30.7	24.0/51.1*	26.0/61.2*
Waist circumference (cm)	83.8 ± 14.7	94.1 ± 15.4*	100.3 ± 13.9*
Central obesity (%)	31.0	50.5*	70.0*
Waist-to-hip ratio	0.80 ± 0.09	0.87 ± 0.11*	0.88 ± 0.10*
Regular tobacco intake (% subjects)	15.0	21.5	7.0
Regular alcohol intake (% subjects)	21.7	27.5	17.4
% with diabetes mellitus	3.1	9.9*	30.2*
% with dyslipidaemia	19.7	24.9	30.6*
% with CVD	4.7	2.6	2.5
% with elevated serum creatinine	0.9	3.0*	7.4*

CVD: cardiovascular disease. *p < 0.001 vs normotensives.

A greater proportion of treated hypertensives had dyslipidaemia (total cholesterol > 6.5 mmol/l, or LDL cholesterol > 4.0 mmol/l, or HDL cholesterol < 1.0 mmol/l in men and < 1.2 mmol/l in women). Only 14.6% of participants smoked. Few participants had pre-existing cardiovascular disease. A greater proportion of treated hypertensives had slightly elevated creatinine concentrations (115–133 μmol/l in men and 107–124 μmol/l in women).

Table 2 shows BP values, BP control rates and the severity of high BP in normotensive participants, hypertensive patients not receiving therapy, and hypertensive patients receiving therapy. Both the hypertensives not receiving therapy and the hypertensives receiving therapy had markedly higher BP values than the normotensive participants, even after adjustments for age. The untreated hypertensives had higher BP values than the treated hypertensive group. In the whole group, 62.3% of participants had normal BP control. In the hypertensives receiving therapy, only 35.9% had normal BP control and hence should have received additional antihypertensive drug therapy; 42.5% of patients with untreated hypertension and 23.9% of patients with treated hypertension had moderate to severe increases in BP.

**Table 2.** BP, Control Of BP And Severity Of BP

	*Normotensives (n = 554)*	*Untreated hypertensives (n = 233)*	*Treated hypertensives (n = 242)*
BP values			
Systolic BP (SBP) (mm Hg)	116 ± 11	151 ± 20*^†^	143 ± 23*
Diastolic BP (DBP) (mm Hg)	77 ± 8	97 ± 10*^†^	89 ± 13*
Age-adjusted SBP (mm Hg)	120 ± 17	148 ± 16*^†^	136 ± 18*
Age-adjusted DBP (mm Hg)	78 ± 11	96 ± 10*^†^	88 ± 11*
Control rates			
% uncontrolled SBP	0	66.1*	51.2*
% uncontrolled DBP	0	85.0*	47.5*
% uncontrolled SBP and DBP	0	100.0*	64.1*
Severity of increased BP			
% with stage I BP^‡^	0	57.5*	40.1*
% with stage II BP^‡^	0	27.0*	14.0*
% with stage III BP^‡^	0	15.5*	9.9*

^‡^See text for definitions. *p < 0.0001 vs normotensives. ^†^*p* < 0.0001 vs treated hypertensives.

Table 3 shows the classes of agents and number of classes of antihypertensive agents used to treat BP in the controlled and uncontrolled hypertensives. Importantly, the majority of patients were receiving diuretic monotherapy. No differences were noted in the classes of agents and number of classes of antihypertensive agents employed in the hypertensives controlled to target BP compared to those not at target BP.

**Table 3.** Drug Therapy In Treated Hypertensives

	*Uncontrolled BP (n = 155)*	*Controlled BP (n = 87)*
% monotherapy	71.0	67.8
% dual therapy	18.0	24.1
% more than 3 agents	11.0	8.1
% diuretic monotherapy	62.6	58.6
% diuretics with other agents	28.4	31.0
Angiotensin-converting enzyme inhibitors (%)	17.4	18.4
Angiotensin receptor blockers (%)	0.7	0
Calcium channel blockers (%)	12.3	14.9
β-adrenergic receptor blockers (%)	2.6	1.2
Others (%)	19.4	17.2

BP: blood pressure.

**Fig. 1.**
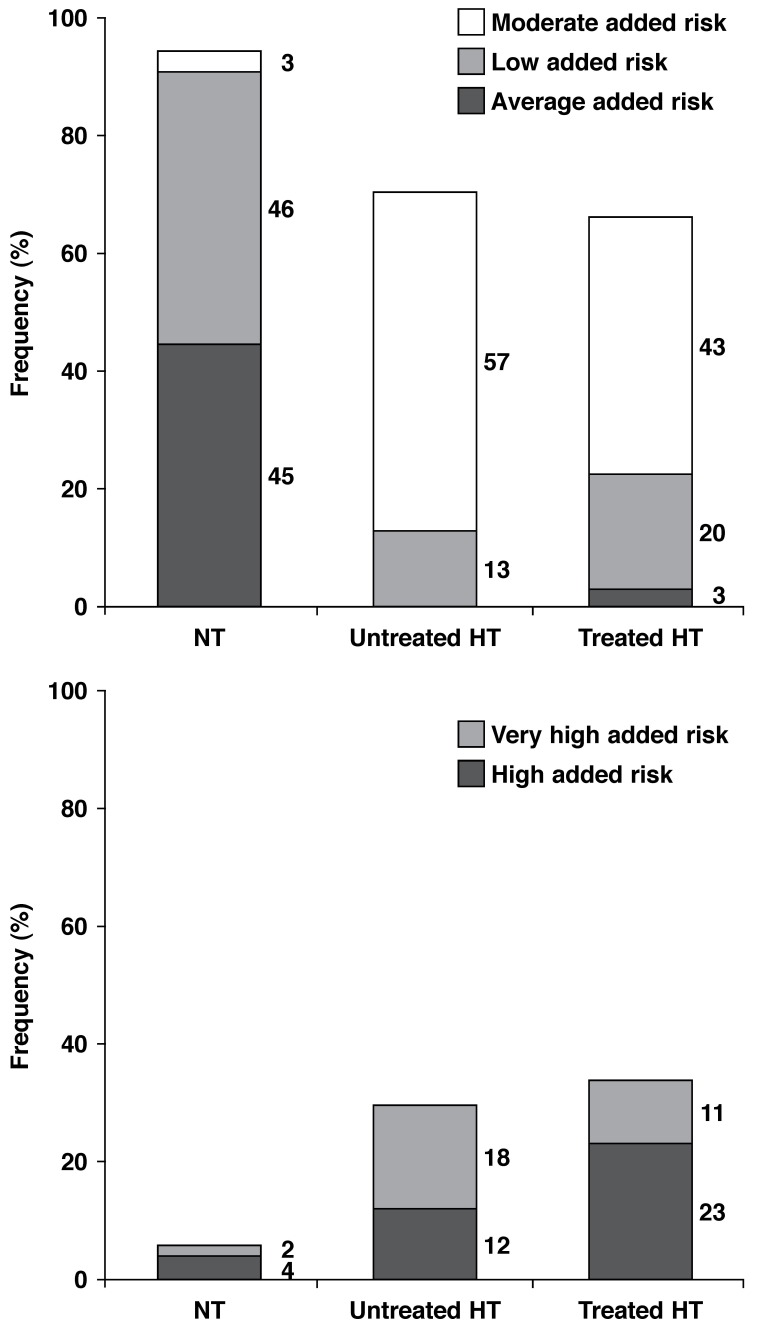
Cardiovascular risk profiles in normotensive, untreated hypertensive and treated hypertensive participants in an urban, developing community of African ancestry. The levels of risk are defined according to current Southern African Hypertension Society^27^ and European Society of Hypertension/European Society of Cardiology^16^ guidelines. High and very high added risk are risk scores that require the initiation of antihypertensive medication. HT: hypertension; NT: normotension.

Fig. 1 shows the distribution of cardiovascular risk profiles in normotensive participants, hypertensive patients not receiving therapy, and hypertensive patients receiving therapy, based on the SAHS and the ESH/ESC guidelines. Importantly, in hypertensives not receiving therapy, 29.6% of participants had either high (~12%) or very high (~18%) added cardiovascular risk. Therefore, based on the SAHS and ESH/ESC guidelines, in the community at large, 6.7% of participants had untreated hypertension together with a high or very high added cardiovascular risk.

With regard to the risk profiles of participants, determined from the WHO/ISH guidelines for low- and middle-income countries, 0.2% of normotensives, 11.2% of untreated hypertensives and 12.8% of treated hypertensives had a > 30% chance of a cardiovascular event in 10 years. These proportions were similar to the proportions noted with a very high added risk, as defined by the SAHS/ESH/ESC guidelines (Fig. 1).

Table 4 shows the factors contributing to cardiovascular risk, as defined by the SAHS/ESH/ESC guidelines in untreated hypertensives. Importantly, while no participants in the lowerrisk category had severe hypertension, DM or cardiovascular disease, of the participants with a high or very high added risk, 52.2% had severe hypertension (36/69 participants), 33.3% had DM (23/69 participants), and 8.7% had an associated cardiovascular condition (6/69). None of the participants with DM had severe hypertension. An increased prevalence of ‘risk’ age (men > 55 years and women > 65 years), dyslipidaemia, and an increased waist circumference was also noted in the high or very high added risk category.

**Table 4.** Characteristics Of Untreated Hypertensives With Different Cardiovascular Risk Categories Based On The Southern African Hypertension Society,^27^ And European Society Of Hypertension/European Society Of Cardiology^16^ Guidelines

	*Untreated hypertensives with cardiovascular risk score of:*	
	*High or very high added*	*Average, low added, or moderate added*
Number	69	164
Age (years)	59.2 ± 14.0*	46.7 ± 15.2
% at risk age	45*	18
% female	59.4	53.7
Body mass index (kg/m^2^)	31.3 ± 6.4*	30.3 ± 7.9
% overweight/obese	26.1/58.0*	23.2/48.2
Waist circumference (cm)	97.0 ± 12.9*	92.9 ± 16.2
Central obesity (%)	67.7*	43.2
Regular tobacco intake (% subjects)	23.2	20.7
Regular alcohol intake (% subjects)	26.1	28.1
% with diabetes mellitus	33.3**	**0**
% with dyslipidaemia	33.3*	21.3
% with CVD	8.7*	**0**
% with elevated serum creatinine	4.4	2.4
Severity of increased BP		
% with stage I BP^†^	34.8*	67.1
% with stage II BP^†^	13.0*	32.9
% with stage III BP^†^	52.1**	**0**

CVD: cardiovascular disease. ^†^See text for definitions. High added indicates 20–30% risk of either a myocardial infarct or a stroke over 10 years. Very high added indicates > 30% risk of either a myocardial infarct or a stroke over 10 years, *p < 0.0001 vs other group. Bold values indicate significant differences between the groups.

In contrast to the factors that characterised a higher versus lower cardiovascular risk in participants, as defined by the SAHS/ESH/ESC guidelines, where no participants in the lower-risk categories had severe hypertension or DM, according to the WHO/ISH guidelines, 9.2 and 7.3% of those with a < 30% chance of a cardiovascular event in 10 years had severe hypertension or DM, respectively. Moreover, when defining risk according to the WHO/ISH guidelines, no differences in either the proportion of participants with general or central obesity, and no differences in mean BMI or waist circumference were noted between those participants with a < 30% or > 30% chance of a cardiovascular event in 10 years (data not shown).

## Discussion

The main findings of the present study are as follows. In an urban, developing community of African ancestry, 22.6% of people had hypertension and were not receiving antihypertensive medication. Importantly, when defining global cardiovascular risk profiles according to the SAHS/ESH/ESC guidelines,^16^,^27^ 6.7% of people have untreated hypertension together with a high or very high added cardiovascular risk. The disturbing cardiovascular risk profiles of untreated hypertensives at a high or very high added risk were characterised by the presence of severe hypertension (systolic BP ≥ 180 mmHg or diastolic BP ≥ 110 mmHg) in ~52%, and the presence of DM in ~33%. In contrast, no untreated hypertensives with lower risk profiles had either severe hypertension or DM.

Although a number of studies have reported on the prevalence of untreated hypertension in a variety of countries,^8^-^13^ including South Africa,^12^,^13^ the present study extends on these studies by reporting on the global cardiovascular risk profiles of untreated hypertensives who are at sufficient risk to consider immediate antihypertensive therapy. In this regard, based on the SAHS/ESH/ESC guidelines,^16^,^27^ 6.7% of these participants (69 of 1 029 participants with a high or very high added risk) were at an absolute 10-year risk of cardiovascular disease of 20–30% (high added risk) or > 30% (very high added risk).^16^ Therefore, over 10 years, a minimum of 1 740 cardiovascular events could occur per 100 000 of the population of African ancestry living in urban, developing communities in South Africa through a lack of antihypertensive drug therapy. This clearly represents a considerable health burden mediated by a lack of drug therapy.

Although in the present study we focused our assessment of global cardiovascular risk prediction based on the SAHS/ESH/ESC guidelines,^16^,^27^ we also calculated global cardiovascular risk from the WHO/ISH risk prediction charts for low- and middleincome countries.^28^ We preferentially focused on the SAHS/ESH/ESC guidelines for a number of reasons. Importantly, WHO/ISH risk charts exclude an analysis that incorporates the risk for obesity. In this regard, we have previously demonstrated that obesity is highly prevalent and independently contributes to cardiovascular damage in urban, developing communities in South Africa.^18^-^24^ Furthermore, WHO/ISH risk charts exclude an assessment of risk in the 20–40-year age category and we have noted that a significant burden of severe hypertension (BP ≥ 180/110 mmHg) exists in participants younger than 40 years of age.

In contrast, the SAHS/ESH/ESC guidelines incorporate the risk of adiposity and allow for an assessment of risk in all age groups.^16^,^27^ Moreover, the recommendation for drug intervention using the WHO/ISH charts has been suggested to be a > 30% risk of a cardiovascular event in 10 years,^28^ which in the population we studied, was likely to have excluded a number of severe hypertensives. Although our focus was on the approach adopted by the SAHS/ESH/ESC guidelines, global cardiovascular risk scores identified using the two approaches were similar, therefore justifying a focus on one approach alone.

In contrast to the South African Health and Demographic Survey conducted in 1998, where only 21% of black hypertensives were receiving antihypertensive medication,^12^ in the present study, approximately 51% of hypertensives were receiving therapy. This clearly represents improved care at a primary-care level in urban, developing communities in South Africa in this ethnic group. This ~51% of hypertensives receiving therapy is a proportion much closer to that obtained in developed nations such as the USA, where 60.4% are receiving treatment,^8^ and also much closer to the proportion of white hypertensives receiving treatment in South Africa in 1998 (~55%).^12^

However, as pointed out in the aforementioned discussion, the caveat to this improved antihypertensive care, compared to previous studies,^12^ is that 6.7% of the remaining untreated hypertensives were at a sufficiently high risk of a cardiovascular event that drug treatment should have been initiated. Moreover, half of these high-risk untreated hypertensives were at a high risk because of the presence of severe hypertension, and a third because of the presence of DM. In contrast, none of the participants in the lower-risk categories had either severe hypertension or DM. These data indicate that it is necessary to introduce programmes at a community level to identify hypertensives with severe hypertension or DM.

Although not a primary aim of this study, it is nevertheless important to comment on the control of BP in treated hypertensives. In this regard, only 35.9% of treated hypertensives were controlled to target BP levels. This is in contrast to the 59.7% of white hypertensives and 48.9% of black hypertensives that are controlled to target BP levels in the USA.^8^ However, the data obtained in the present study are in keeping with the 33% of hypertensive peri-urban black South Africans that achieved BP control in alternative studies.^14^,^15^

A possible explanation for the low BP control rates in the treated hypertensives in the present study is therapeutic inertia.^31^ Indeed, ~70% of treated hypertensives were receiving monotherapy only, and it is presently well accepted that monotherapy does not achieve BP control in the majority of hypertensives. Importantly however, 76% of treated hypertensives had BP levels < 159/99 mmHg, and therefore, to achieve BP control in all treated hypertensives may not require a considerable increase in drug therapy.

Due to the high prevalence of obesity, a limitation of the present study was our inability to appropriately assess target-organ changes from electrocardiography. This could therefore have resulted in us underestimating the extent of cardiovascular risk in the untreated hypertensives and hence would have biased against the outcomes of the study. In a sub-study conducted with echocardiography and electrocardiography, we are presently attempting to identify the most effective electrocardiographic criteria that may be employed to identify left ventricular hypertrophy in obese individuals of African ancestry. In addition, because we had no method of validating the answers of questions related to adherence to antihypertensive therapy, our study was limited in that we were unable to identify reasons for a lack of effective treatment in the hypertensives. However, notwithstanding the importance of such a question, this was not the primary aim of our study.

## Conclusion

In urban, developing communities of African ancestry, 6.7% of people may have untreated hypertension together with an overall cardiovascular risk profile that requires drug therapy. This could translate, over 10 years, into 1 740 cardiovascular events per 100 000 of the population of African ancestry living in urban, developing communities in South Africa, due to a lack of antihypertensive drug therapy. This clearly represents a considerable health burden that can only be rectified by introducing programmes at a community level to identify hypertensives that require drug therapy. In this regard, the majority of these individuals had a high risk because of either the presence of severe hypertension or DM.
